# Synthesis and Biological Activity Evaluation of Novel *β*-Substituted Nitromethylene Neonicotinoid Analogues

**DOI:** 10.3390/molecules170910014

**Published:** 2012-08-24

**Authors:** Baozhu Wang, Jiagao Cheng, Zhiping Xu, Xiaoyong Xu, Xusheng Shao, Zhong Li

**Affiliations:** Shanghai Key Laboratory of Chemical Biology, School of Pharmacy, East China University of Science and Technology, P.O. Box 544, 130 Meilong Road, Shanghai 200237, China

**Keywords:** neonicotinoid analogues, new design strategy, thiocyanogen, insecticidal activities

## Abstract

The structure-based design and synthesis of a series of novel neonicotinoid analogues are described. The novel neonicotinoid analogues were designed based upon the reaction of enamine derivatives with electron-withdrawing *β*-substituents with electrophilic thiocyanogen reagents. These compounds were characterized by spectroscopic methods. Bioassays indicated that some of the synthesized compounds exhibited excellent bioactivity against cowpea aphids (*Aphis craccivora*). The LC_50_ values of compounds **7**, **9**, **12**, **13**, **15**, **17**, **19**, **20** and commercial imidacloprid were 0.01567, 0.00974, 0.02494, 0.01893, 0.02677, 0.01778, 0.0220, 0.02447 and 0.03502 mmol L^−1^, respectively, which suggested that they could be used as leads for future development of new insecticides.

## 1. Introduction

In recent years, neonicotinoid insecticides have been the fastest growing class of insecticides in modern crop protection, with widespread use against a broad spectrum of sucking and certain chewing pests [1,2,3,4,5]. Significant progress had been obtained on the chemistry of the insecticidal nicotinic agonists and the chemical biology of the nicotinic acetylcholine receptors (nAChRs), that is, chemorational approaches including physicochemical considerations, metabolism, resistance mechanisms, and chemical and structural biology aspects potentially expediting receptor structure-guided insecticide design [6,7,8,9,10,11,12].

**Figure 1:**
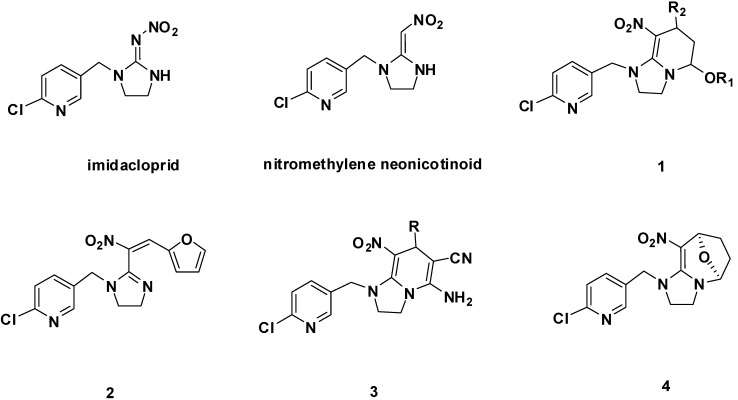
.Structures of imidacloprid neonicotinoids and structurally related analogues.

Our past endeavors in this field have focused on systematic pharmacophore modifications of nitromethylene neonicotinoids that appeared to capture the “bioactive” conformation (Figure 1). Several types of nitromethylene neonicotinoids with the *cis*-configuration were presented; that is, neonicotinoids **1** with a tetrahydropyridine fixed *cis*-configurations; neonicotinoids **2** with bulky group fixed *cis*-configurations, neonicotinoids **3** with *cis*-configuration constructed by aza-Diels-Alder reactions, and divalent and oxa-bridged neonicotinoids **4** constructed from dialdehydes (Figure 1). Bioassays indicated that the synthesized *cis* compounds **1**–**4** exhibited excellent bioactivity against cowpea aphids [13,14,15,16]. According to our knowledge, the nitro groups in all commercialized neonicotinoids have a *trans* configuration, on which three proposals for mode of action are based [17]. The high bioactivity of compounds **1**–**4** implies that neonicotinoids in the *cis* configuration might bind to the receptor in a different way [13]. These efforts accordingly encourage the discovery of “superneonicotinoids” effective for imidacloprid-resistant pests and lepidoptera species.

**Figure 2:**
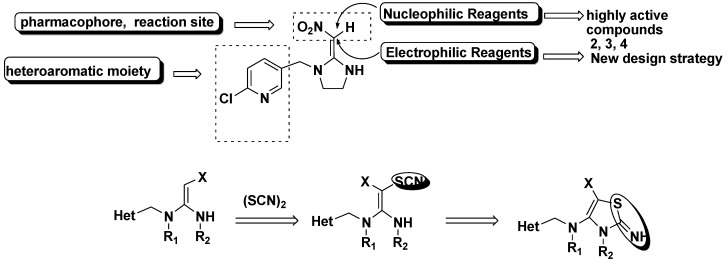
.Structures of imidacloprid neonicotinoids and its structurally related analogues.

The structure derivative method through the organic reaction is another important tool for systematic pharmacophore modifications [18]. It had been theorized that the nitroenamine entities in these high active compounds play important roles for specific binding to the desired insect nAChRs site [19,20,21,22]. Encouraged by these reports, the modifications of nitroenamine entities to fix its configuration through organic reactions has attracted our attention. The chemistry of enamines, including push–pull enamines bearing an additional electron-acceptor group at the *β*-position, has been extensively developed, mainly as a result of electrophilic reactions that proceed predominantly at the *β*-position [23,24,25]. Electrophilic modification, followed by a set of transformations, have made enamines an integral part of synthetic organic chemistry methodology [26]. Additionally, electron-acceptor groups can participate in chemical transformations. According to this knowledge, a series of prototype compounds with extended N-substituted (thiocyanogen) substituents were designed in this paper (Figure 2). The choice of the thiocyanogen was predominantly governed by its traits as electrophilic reagent, and mainly by its ability to easily cyclize with the NH group in the imidazoline ring to fix the electron-withdrawing group in the *cis* position (Figure 2) [27].

## 2. Results and Discussion

### 2.1. Synthetic Chemistry

**Scheme 1:**
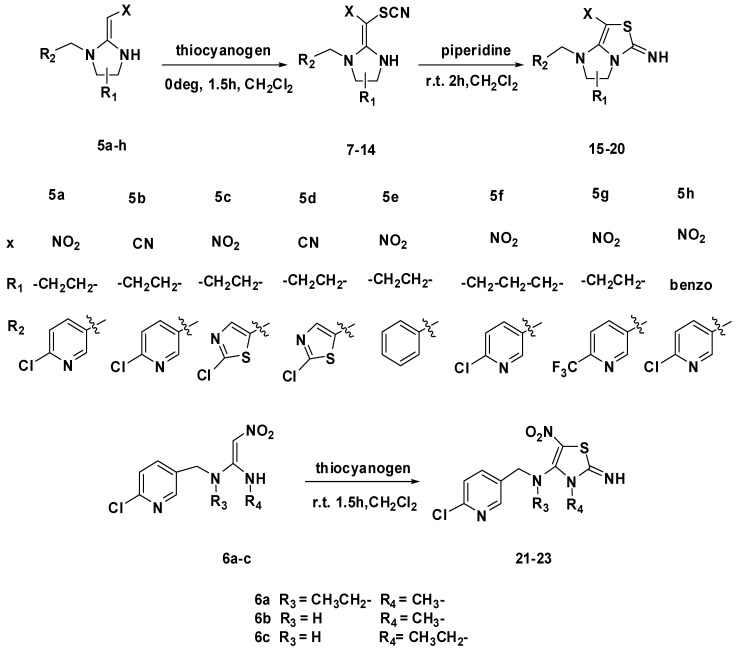
.Synthetic route for the title compounds **7**–**23**.

**Figure 3:**
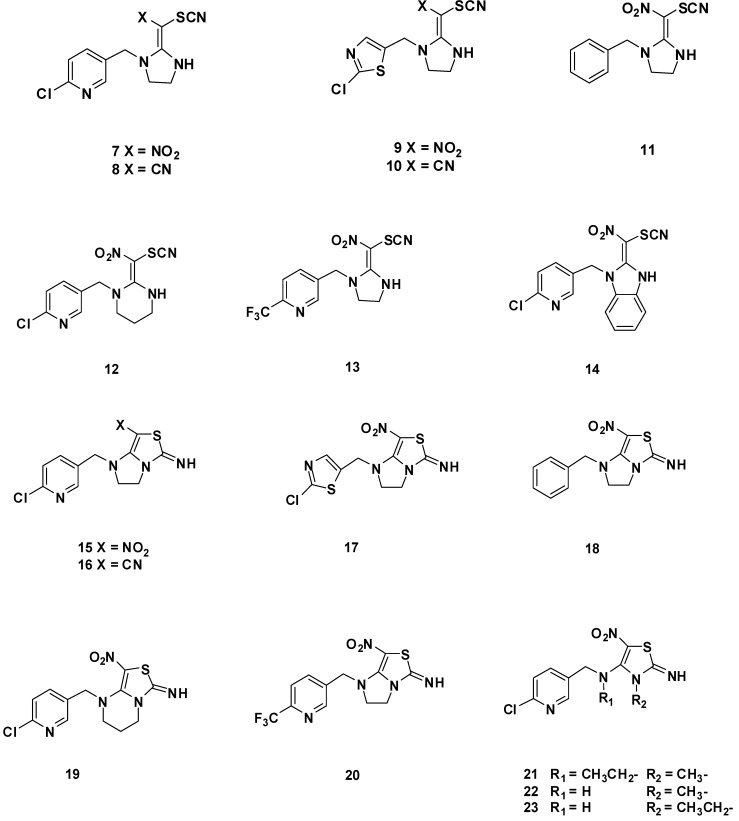
Chemical structures of novel neonicotinoid analogues under study.

The synthetic route of the target compounds is summarized in Scheme 1. The chemical structures of the novel neonicotinoid analogues under study are depicted in Figure 3. The preparation of the designed compounds began with the common intermediates **5a**–**h** and **6a**–**c** which were synthesized according to the method of Kagabu *et al.* [19]. In the reaction of **5a**–**h** with thiocyanogen at 0 °C for 1.5 h, the corresponding *β*-thiocyanated products **7**–**14** were produced in 63%–82% yields. In this procedure, **5a** appears to produce a mixture of **7** and cyclization product **15**. Similarly, **5c** and **5f** also gave mixtures of aminothiazole and the relevant cyclization product. Compounds **7**, **9** and **12** were synthesized by reducing the reaction time and reaction temperature without forming cyclization products. Then, these compounds **7**–**9** and **11**–**13** were converted quantitatively into the corresponding cyclization products, **15**–**20**, by stirring at room temperature in dichloromethane solution containing 0.1% piperidine as catalyst. Unexpectedly, **10** and **14** could not be converted into the corresponding cyclization products. The substrates **6a**–**c** yielded only the corresponding cyclization products **21**–**23** respectively under the same conditions. Whereas configuration of compounds **7**–**14** and **21**–**23** cannot be determined easily by standard ^1^H- and ^13^C-NMR techniques, the compounds **15**–**20** obtained from the reaction system are considered to be produced with retention of the configuration of the preformed enamines with electron-withdrawing *β*-substituents, followed by catalyzed cyclization. The structures of the title compounds were well characterized by ^1^H-NMR, ^13^C-NMR and HRMS.

### 2.2. Biological Activities

The insecticidal activities of the designed compounds against cowpea aphids are shown in Table 1. Most of the title compounds showed excellent insecticidal activities against cowpea aphids. For example, the LC_50_ values of compounds **7**, **9**, **12**, **13**, **15**, **17**, **19**, and **20** were 0.01567, 0.00974, 0.02494, 0.01893, 0.02677, 0.01778, 0.0220 and 0.02447 mmol L^−1^, respectively, whereas that of imidacloprid was only 0.03502 mmol L^−1^ [16]. Activities varied significantly depending upon the patterns of substituents on the enamine and imidazole ring. It was observed that compounds with NO_2_ substituents (**7**, **15**, **9** and **17**) demonstrated better activities than that with CN substituents (**8**, **10** and **16**). Generally, the imidazolidine substituents appear to be the important factor for insecticidal activities. A benzene substituent (compound **14**) resulted in complete inactivity. Moreover, the cyclic addition of SCN substituents with the imidazolidine ring is unfavourable for high activities from the data analysis present of compounds **7**, **9**, **15** and **17**.

**Table 1:** .Insecticidal activities of compounds **7**–**23** against cowpea aphids (*Aphis craccivora*).

Compounds	Mortality (%, 500mg L^−1^)	LC_50_ (mmol L^−1^)
**7**	100	0.01567
**8**	34.6	nt ^a^
**9**	100	0.00974
**10**	0	nt
**11**	23.98	nt
**12**	100	0.02494
**13**	100	0.01893
**14**	87.82	nt
**15**	100	0.02677
**16**	32.4	nt
**17**	100	0.01778
**18**	16.19	nt
**19**	100	0.02200
**20**	100	0.02447
**21**	57.6	nt
**22**	100	0.11839
**23**	50.79	nt

^a^ Not tested.

## 3. Experimental

### 3.1. General

Melting points (mp) were recorded on a Büchi B540 apparatus (Büchi Labortechnik AG, Flawil, Switzerland) and are uncorrected. ^1^H-NMR and ^13^C-NMR spectra were recorded on a Bruker AM-400 (400 MHz) spectrometer with CDCl_3_ or DMSO-*d_6_* as the solvent and TMS as the internal standard. Chemical shifts are reported in δ (parts per million) values. High-resolution mass spectra were recorded under electron impact (70 eV) condition using a MicroMass GCT CA 055 instrument. Analytical thin-layer chromatography (TLC) was carried out on precoated plates (silica gel 60 F_254_), and spots were visualized with ultraviolet (UV) light. The compounds **5a**–**h** and **6a**–**c** were synthesized according to the method of Kagabu *et al.* [19]. Unless otherwise noted, reagents and solvents were used as received from commercial suppliers. Yields were not optimized. All reactions were carried out under a protective atmosphere of drying nitrogen or utilizing a calcium chloride drying tube.

#### 3.1.1. General Synthetic Procedure for **7–14**

To a solution of **5a**–**g** (10 mmol) in dichloromethane (100 mL), a thiocyanogen (10 mmol) solution in dichloromethane (50 mL), which was previously prepared from lead thiocyanate and bromine at −15 °C, was added dropwise with stirring at −15 °C and then the mixture was stirred for 1.5 h at 0 °C. The reaction mixture was washed with cold water (3 × 50 mL) and brine (50 mL), and dried with sodium sulfate. The solvent was removed to give a crude solid, which was recrystallized from a mixture of dichloromethane/ether (2:1, v/v) to give **7**–**14**.

*2-Chloro-5-((2-(nitro(thiocyanato)methylene)imidazolidin-1-yl)methyl)pyridine* (**7**). Yield: 92%, m.p.: 130.6–132.1 °C; ^1^H-NMR (DMSO-*d_6_*): *δ* 3.76 (br.s, 4H), 4.77 (s, 2H), 7.55 (d, *J* = 8.4 Hz, 1H), 7.86 (dd, *J_1_* = 2.6 Hz, *J_2_* = 8.4 Hz, 1H), 8.42 (d, *J* = 2.0 Hz, 1H), 9.43 (s, 1H) ppm; ^13^C-NMR (DMSO-*d_6_*): *δ* 162.6, 149.9, 149.1, 139.1, 131.8, 124.7, 112.3, 87.5, 50.7, 50.2, 42.7; HRMS (ES+) calcd for C_11_H_10_^35^ClN_5_O_2_S (M+H)^+^: 312.0244. Found: 312.0330. Calcd for C_11_H_10_^37^ClN_5_O_2_S (M+H)^+^: 314.0214 Found: 314.0284.

*2-(1-((6-Chloropyridin-3-yl)methyl)imidazolidin-2-ylidene)-2-thiocyanatoacetonitrile* (**8**). Yield: 63%, m.p.: 118.2–119.7 °C; ^1^H-NMR (DMSO-*d_6_*): *δ* 3.51–3.56 (m, 2H), 3.66–3.70 (m, 2H), 4.79 (s, 2H), 7.54 (d, *J* = 8.4 Hz, 1H), 7.78–7.80 (m, 1H), 8.11 (s, 1H), 8.36 (d, *J* = 2.4 Hz, 1H) ppm; ^13^C-NMR (DMSO-*d_6_*): *δ* 164.9, 150.0, 149.2, 139.2, 132.1, 124.8, 122.1, 113.8, 51.6, 47.4, 41.5, 31.2; HRMS (EI+) calcd for C_12_H_10_^35^ClN_5_S (M^+^): 291.0345. Found: 291.0344. Calcd for C_12_H_10_^37^ClN_5_S (M^+^): 293.0316 Found: 293.0318.

*1-((2-Chlorothiazol-5-yl)methyl)-2-(nitro(thiocyanato)methylene)imidazolidine* (**9**). Yield: 75%, m.p.: 85.1–86.3 °C; ^1^H-NMR (DMSO-*d_6_*): *δ* 3.69–3.78 (m, 4H), 4.84 (s, 2H), 7.73 (s, 1H), 9.40 (s, 1H) ppm; ^13^C-NMR (DMSO-*d_6_*): *δ* 162.2, 151.6, 141.8, 135.9, 112.1, 87.3, 50.1, 46.3, 42.6; HRMS (ES+) calcd for C_9_H_8_^35^ClN_5_O_2_S_2_ (M+H)^+^: 317.9808. Found: 317.9890. Calcd for C_9_H_8_^37^ClN_5_O_2_S_2_ (M+H)^+^: 319.9778 Found: 319.9860.

*2-(1-((2-Chlorothiazol-5-yl)methyl)imidazolidin-2-ylidene)-2-thiocyanatoacetonitrile* (**10**). Yield: 52%, m.p.: 120.3–122.2 °C; ^1^H-NMR (DMSO-*d_6_*): *δ* 3.30–3.70 (m, 4H), 4.93 (s, 2H), 7.70 (s, 1H), 8.17 (s, 1H) ppm; ^13^C-NMR (DMSO-*d_6_*): *δ* 164.5, 151.3, 141.6, 135.8, 122.0, 113.5, 51.0, 43.0, 41.5, 31.8; HRMS (ES−) calcd for C_10_H_8_^35^ClN_5_S (M−H)^+^: 295.9910. Found: 295.9829. Calcd for C_10_H_8_^37^ClN_5_S (M−H)^+^: 297.9880 Found: 297.9803.

*1-Benzyl-2-(nitro(thiocyanato)methylene)imidazolidine* (**11**). Yield: 82%, m.p.: 102.3–104.2 °C; ^1^H-NMR (DMSO-*d_6_*): *δ* 3.72 (s, 4H), 4.75 (s, 2H), 7.32–7.42 (m, 5H), 9.40 (s, 1H) ppm; ^13^C-NMR (DMSO-*d_6_*): *δ* 162.3, 136.1, 129.2, 128.1, 127.6, 112.3, 86.9, 52.7, 50.3, 42.6; HRMS (ES+) calcd for C_12_H_12_N_4_O_2_S (M+H)^+^: 277.0681. Found: 277.0750.

*1-((6-Chloropyridin-3-yl)methyl)-2-(nitro(thiocyanato)methylene)-hexahydropyrimidine* (**12**). Yield: 70%, m.p.: 158.1–160.3 °C; ^1^H-NMR (DMSO-*d_6_*): *δ* 2.14 (t, *J* = 5.2, 2H), 3.38 (t, *J* = 5.6 Hz, 2H), 3.85 (t, *J* = 5.6 Hz, 2H), 4.06 (s, 2H), 7.57 (d, *J* = 8.0 Hz, 1H), 7.91 (dd, *J_1_* = 8.4 Hz, *J_2_* = 2.8 Hz, 1H), 8.45 (d, *J* = 2.4 Hz, 1H) ppm; ^13^C-NMR (DMSO-*d_6_*): *δ* 164.7, 158.9, 150.2, 146.2, 140.1, 131.0, 124.5, 106.5, 56.8, 47.2, 45.1, 19.1; HRMS (ES+) calcd for C_12_H_12_^35^ClN_5_O_2_S (M+H)^+^: 326.0400. Found: 326.0477. Calcd for C_12_H_12_^37^ClN_5_ O_2_S (M+H)^+^: 328.0371 Found: 328.0465.

*5-((2-(Nitro(thiocyanato)methylene)imidazolidin-1-yl)methyl)-2-(trifluoromethyl)pyridine* (**13**). Yield: 82%, m.p.: 140.1–142.3 °C; ^1^H-NMR (DMSO-*d_6_*): *δ* 3.76 (s, 4H), 3.38 (t, *J* = 5.6 Hz, 2H), 4.85 (s, 2H), 7.91 (d, *J* = 8.0 Hz, 1H), 8.04 (s, 1H), 8.75 (s, 1H), 9.45 (s, 1H) ppm; ^13^C-NMR (DMSO-*d_6_*): *δ* 162.7, 149.3, 137.2, 136.6, 121.1, 112.3, 87.7, 50.9, 50.8, 42.8; HRMS (ES+) calcd for C_12_H_10_F_3_N_5_O_2_S (M+H)^+^: 345.0507. Found: 345.0617.

*(E-1-((6-Chloropyridin-3-yl)methyl)-2-(nitro(thiocyanato)methylene)-2,3-dihydro-1H-enzo[d]imid-azole* (**14**). Yield: 74%, m.p.: 125.6–127.2 °C; ^1^H-NMR (DMSO-*d_6_*): *δ* 4.79 (s, 2H), 7.39–7.7 (m, 6H), 8.35 (d, *J* = 2.4 Hz, 1H) ppm; ^13^C-NMR (DMSO-*d_6_*): *δ* 150.1, 148.7, 148.6, 138.5, 132.5, 131.3, 130.4, 126.0, 125.4, 124.8, 113.8, 112.2, 112.0, 86.0, 55.4, 47.2; HRMS (ES−) calcd for C_10_H_8_^35^ClN_5_S (M−H)^+^: 358.0244. Found: 358.0130. Calcd for C_10_H_8_^37^ClN_5_S (M−H)^+^: 360.0214. Found: 297.0166.

#### 3.1.2. General Synthetic Procedure for **15–20**

The solution of **7**–**9** and **11**–**13** (10 mmol) in dichloromethane (50 mL) containing 0.1% piperidine was stirred for 2 h at room temperature. After removal of the solvent, the residue was recrystallized from dichloromethane to give **15**–**20** in quantitative yields.

*1-((6-Chloropyridin-3-yl)methyl)-7-nitro-2,3-dihydroimidazo*[1,2-c]*thiazol-5(1H)-imine* (**15**). Yield: 93%, m.p.: 136.4–138.5 °C; ^1^H-NMR (DMSO-*d_6_*): *δ* 3.85 (s, 2H), 4.08 (s, 2H), 5.27 (s, 2H), 7.58 (br.s, 1H), 7.91–7.92 (m, 1H), 8.47 (s, 1H), 8.91 (br.s, 1H) ppm; ^13^C-NMR (DMSO-*d_6_*): *δ* 150.6, 150.1, 149.7, 139.8, 132.0, 124.8, 99.7, 55.7, 49.4, 41.8; HRMS (ES+) calcd for C_11_H_10_^35^ClN_5_O_2_S (M+H)^+^: 312.0244. Found: 312.0321. Calcd for C_11_H_10_^37^ClN_5_O_2_S (M+H)^+^: 314.0279. Found: 314.0284.

*1-((6-Chloropyridin-3-yl)methyl)-5-imino-1,2,3,5-tetrahydroimidazo*[1,2-c]*thiazole-7-carbonitrile* (**16**). Yield: 82%, m.p.: 201.5–202.3 °C; ^1^H-NMR (DMSO-*d_6_*): *δ* 3.87 (s, 4H), 4.61 (s, 2H), 7.41 (d, *J* = 8.0 Hz, 1H), 7.73–7.76 (m, 1H), 8.38 (d, *J* = 2.0 Hz, 1H) ppm; ^13^C-NMR (DMSO-*d_6_*): *δ* 155.2, 153.0, 152.0, 149.3, 138.9, 129.1, 125.0, 114.7, 53.2, 47.8, 47.2, 41.0; HRMS (ES+) calcd for C_12_H_10_^35^ClN_5_S (M+H)^+^: 292.0345. Found: 292.0430. Calcd for C_12_H_10_^37^ClN_5_S (M+H)^+^: 294.0316. Found: 294.0410.

*1-((2-Chlorothiazol-5-yl)methyl)-7-nitro-2,3-dihydroimidazo*[1,2-c]*thiazol-5(1H)-imine* (**17**). Yield: 78%, m.p.: 150.3–151.4 °C; ^1^H-NMR (DMSO-*d_6_*): *δ* 3.81–4.16 (m, 4H), 5.34 (s, 2H), 7.77 (s, 1H), 8.96 (s, 1H) ppm; ^13^C-NMR (DMSO-*d_6_*): *δ* 152.3, 150.0, 142.0, 136.0, 99.9, 55.5, 45.2, 41.7; HRMS (EI+) calcd for C_9_H_8_ClN_5_O_2_S_2_ (M^+^): 316.9808. Found: 316.9807.

*1-Benzyl-7-nitro-2,3-dihydroimidazo*[1,2-c]*thiazol-5(1H)-imine* (**18**). Yield: 74%, m.p.: 82.3–84.4 °C; ^1^H-NMR (DMSO-*d_6_*): *δ* 3.81–4.06 (m, 4H), 5.26 (s, 2H), 7.32–7.43 (s, 1H), 8.96 (s, 1H) ppm; ^13^C-NMR (DMSO-*d_6_*): *δ* 150.2, 136.4, 129.2, 128.3, 55.5, 52.3, 41.6; HRMS (ES+) calcd for calcd for C_12_H_12_N_4_O_2_S (M+H)^+^: 277.0681. Found: 277.0746.

*1-((6-Chloropyridin-3-yl)methyl)-8-nitro-1,2,3,4-tetrahydrothiazolo*[3,4-a]*pyrimidin-6-imine* (**19**). Yield: 65%, m.p.: 165.3–167.4 °C; ^1^H-NMR (DMSO-*d_6_*): *δ* 2.01 (t, *J* = 5.2 Hz, 2H), 3.30 (t, *J* = 5.6 Hz, 2H), 3.73 (t, *J* = 5.6 Hz, 2H), 4.90 (s, 2H), 7.53 (d, *J* = 8.4 Hz, 1H), 7.92 (dd, *J_1_* = 8.4 Hz, *J_2_* = 2.4 Hz, 1H), 8.43 (d, *J* = 2.0, 1H), 9.19 (s, 1H) ppm; ^13^C-NMR (DMSO-*d_6_*): *δ* 153.5, 150.5, 150.0, 149.8, 139.9, 131.9, 124.4, 103.2, 56.5, 47.3, 41.6, 19.3; HRMS (ES+) calcd for C_12_H_12_^35^ClN_5_O_2_S (M+H)^+^: 326.0400. Found: 326.0476. Calcd for C_12_H_12_^37^ClN_5_O_2_S (M+H)^+^: 328.0371. Found: 328.0473.

*7-Nitro-1-((6-(trifluoromethyl)pyridin-3-yl)methyl)-2,3-dihydroimidazo*[1,2-c]*thiazol-5(1H)-imine* (**20**). Yield: 88%, m.p.: 148.2–149.3 °C; ^1^H-NMR (DMSO-*d_6_*): *δ* 3.85 (t, *J* = 8.2 Hz, 2H), 4.10 (t, *J* = 9.2 Hz, 2H), 5.36 (s, 2H), 7.94 (d, *J* = 8.4 Hz, 1H), 8.13 (d, *J* = 8.4 Hz, 1H), 8.81(s, 1H), 8.94 (s, 1H) ppm; ^13^C-NMR (DMSO-*d_6_*): *δ* 151.0, 149.8, 137.8, 136.8, 121.1, 56.0, 50.0, 41.8; HRMS (ES+) calcd for C_12_H_10_F_3_N_5_O_2_S (M+H)^+^: 346.0507. Found: 346.0634.

#### 3.1.3. General Synthetic Procedure for **21–23**

To a solution of **6a**–**c** (10 mmol) in dichloromethane (50 mL) was added with stirring at −15 °C a dichloromethane solution of thiocyanogen (10 mmol) which had previously prepared from lead thiocyanate and bromine at −15 °C, and then the mixture was stirred for 1.5 h at room temperature. The reaction mixture was washed with cold water (3 × 50 mL) and brine (50 mL) and then dried with sodium sulfate. The solvent was evaporated *in vacuo*, and fractional precipitation of the residue from dichloromethane afforded **21**–**23**.

*N-((6-Chloropyridin-3-yl)methyl)-N-ethyl-2-imino-3-methyl-5-nitro-2,3-dihydrothiazol-4-amine* (**21**). Yield: 84%, m.p.: 120.3–121.4 °C; ^1^H-NMR (DMSO-*d_6_*): *δ* 1.14 (t, *J* =7.2 Hz, 3H), 3.17 (d, *J* = 7.2 Hz, 2H), 3.40 (s, 3H), 4.57 (s, 2H), 7.54 (d, *J* = 8.0 Hz, 1H), 7.92–7.95 (m, 1H), 8.49 (d, *J* = 2.0 Hz, 1H) ppm; ^13^C-NMR (DMSO-*d_6_*): *δ* 165.1, 150.7, 150.5, 140.9, 131.8, 124.8, 118.2, 51.9, 46.8, 36.1, 13.9; HRMS (EI+) calcd for C_12_H_14_ClN_5_O_2_S (M^+^): 327.0557. Found: 327.0559.

*N-((6-Chloropyridin-3-yl)methyl)-2-imino-3-methyl-5-nitro-2,3-dihydrothiazol-4-amine* (**22**). Yield: 82%, m.p.: 116.4–118.4 °C; ^1^H-NMR (DMSO-*d_6_*): *δ* 3.28 (s, 3H), 5.37 (s, 2H), 7.80 (s, 1H) ppm; ^13^C-NMR (DMSO-*d_6_*): *δ* 152.1, 142.5, 141.2, 42.5, 34.6, 33.3; HRMS (ES−) calcd for C_8_H_8_^35^ClN_5_O_2_S_2_(M−H)^+^: 303.9808. Found: 303.9716. calcd for C_8_H_8_^37^ClN_5_O_2_S_2_ (M−H)^+^: 305.9778. Found: 305.9703.

*N-((6-Chloropyridin-3-yl)methyl)-3-ethyl-2-imino-5-nitro-2,3-dihydrothiazol-4-amine* (**23**). Yield: 78%, m.p.: 115.4–117.2 °C; ^1^H-NMR (DMSO-*d_6_*): *δ* 1.23 (t, *J* = 7.2 Hz, 3H), 3.66 (q, *J* = 7.2 Hz, 2H), 5.34 (s, 2H), 7.81 (s, 1H); ^13^C-NMR (DMSO-*d_6_*): *δ* 152.1, 141.2, 134.7, 42.7, 42.1, 15.7; HRMS (ES−) calcd for C_9_H_10_^35^ClN_5_O_2_S_2_ (M-H)^+^: 317.9964. Found: 317.9870. calcd for C_9_H_10_^37^ClN_5_O_2_S_2_ (M−H)^+^: 319.9935. Found: 319.9836.

### 3.2. Biological Assay

All compounds were dissolved in acetone and diluted with water containing Triton X-100 (0.1 mg L^−1^) to obtain series concentrations of 500.0, 250.0, 125.0 mg L^−1^ and others for bioassays. Pea aphids (*Aphis cracci*v*ora*) were dipped according to our previously reported procedure [13,23]. Tender shoots of soybean with 40-60 healthy apterous adults were dipped in diluted solutions of the chemicals containing Triton X-100 (0.1 mg L^−1^) for 5 s, the superfluous fluid was removed, and the shoots were placed in the conditioned room (25 ± 1 °C, 50% RH). Water containing Triton X-100 (0.1 mg L^−1^) was used as control. Mortality was assessed after 24 h. Each treatment had three repetitions, and the data were adjusted and subjected to probit analysis as before.

## 4. Conclusions

In conclusion, a series of a novel class of neonicotinoids, in which the common nitromethylene pharmacophore was substituted by electrophilic thiocyanogen reagents, were designed and synthesized. Most of the compounds exhibited excellent insecticidal activities against cowpea aphids (*A. craccivora*), which implied that reaction of nitromethylene with the electrophilic system to obtain novel neonicotinoids analogues with high activities was feasible.
